
JOURNAL INFORMATION
==============================
NLM Title Abbreviation: J Orthop Traumatol
Journal ID: J Orthop Traumatol
NLM Journal ID: 101090931

Journal of Orthopaedics and Traumatology : Official Journal of the Italian Society of Orthopaedics and Traumatology

ISSN: 1590-9921
EISSN: 1590-9999
Publisher: Springer

ARTICLE INFORMATION
==============================
PMCID: PMC4417870
DOI: 10.1007/s10195-013-0231-5
Article ID: 231
Article version: 1
Subjects: Acknowledgement to Referees

2012 Scientific Referees

Electronic publication date: 2013 Feb 23
Print publication date: 2013 Mar
Volume: 14
Issue: 1
First page: 75
Last page: 76
Copyright: © The Author(s) 2013
License: This article is distributed under the terms of the Creative Commons Attribution License which permits any use, distribution, and reproduction in any medium, provided the original author(s) and the source are credited.
License URL: https://creativecommons.org/licenses/by/4.0/

issue-copyright-statement: © The Author(s) 2013

==============================

The quality of the *Journal of Orthopaedics and Traumatology* depends on the qualified and regular collaboration of renowned scientists who devoted their time to constructively review the submitted articles. We are indebted to the following experts who reviewed papers which completed the peer-reviewing process within 2012.

Roberto Adani (Verona, Italy)
Ezio Adriani (Rome, Italy)
Mustafa Alnaib (Gateshead, UK)
Annunziato Amendola (Iowa, USA)
Antonio Andreacchio (Turin, Italy)
Alessandro Aprato (Turin, Italy)
Stefano Artiaco (Turin, Italy)
Francesco Atzori (Turin, Italy)
Angelo Aulisa (Rome, Italy)
Andrea Baldini (Florence, Italy)
Massimo Balsano (Vicenza, Italy)
Giovanni Barbanti Brodano (Bologna, Italy)
Vittorio Bellotti (Barcelona, Spain)
Karl Benedetto (Feldkirch, Austria)
Alexander Berth (Magdeburg, Germany)
Celeste Bertone (San Donato Milanese, Italy)
Giuseppe Bianchi (Bologna, Italy)
Amin Sadegh Bigham (Shahrekor, Iran)
Kristian Bjorgul (Fredrikstad, Norway)
Silvio Luigi Boero (Genoa, Italy)
Andrea Borgo (Padua, Italy)
Mario Borroni (Rozzano, Italy)
Elena Maria Brach del Prever (Turin, Italy)
Sergio Brambilla (Milan, Italy)
Matteo Cadossi (Bologna, Italy)
Filippo Calderazzi (Parma, Italy)
Domenico Campanacci (Florence, Italy)
Fabrizio Campi (Cattolica, Italy)
Marco Cassini (Legnago, Italy)
Maurizio Catagni (Lecco, Italy)
Andrea Celli (San Donato Milanese, Italy)
Riccardo Ciatti (Rome, Italy)
Norberto Confalonieri (Milan, Italy)
Luigi Conforti (Turin, Italy)
Maria Cristina D'Agostino (Rozzano, Italy)
Ferdinando Da Rin (Cortina d'Ampezzo, Italy)
Walter Daghino (Turin, Italy)
Carlo Dall'Oca (Verona, Italy)
Umberto De Bellis (Milan, Italy)
Orazio De Lucia (Milan, Iatly)
Luigi De Palma (Ancona, Italy)
Angelo Dettoni (Turin, Italy)
Shabir A. Dhar (Srinagar, India)
Alberto Di Martino (Rome, Italy)
Vitantonio Di Gennaro (Bologna, Italy)
Davide Maria Donati (Bologna, Italy)
Onofrio Donzelli (Bologna, Italy)
Francesco Falciglia (Rome, Italy)
Pasquale Farsetti (Rome, Italy)
Francesco Fauci (Cattolica, Italy)
Andrea Ferretti (Rome, Italy)
Florian Fischer (Milan, Italy)
Maurizio Fontana (faenza, Italy)
Matteo Formica (Genoa, Italy)
Olimpio Galasso (Catanzaro, Italy)
Eduardo García-Rey (Madrid, Spain)
Alessandro Gasbarrini (Bologna, Italy)
Giorgio Gasparini (Catanzaro, Italy)

Giuseppe Giannicola (Rome, Italy)
Federico Grassi (Novara, Italy)
Enrico Guerra (Bologna, Italy)
Tetsuo Hagino (Kofu Yamanashi, Japan)
Massimo Innocenti (Florence, Italy)
Ernesto Ippolito (Rome, Italy)
Nicola Ivaldo (Acqui Terme, Italy)
Ozkan Kose (Antalya, Turkey)
Antonios Kouzelis (Patras, Greece)
Franco Maria Lavini (Verona, Italy)
Danilo Leonetti (Bologna, Italy)
Giandomenico Logroscino (Rome, Italy)
Giovanni Lovisetti (Menaggio, Italy)
Giannicola Lucidi (Rimini, Italy)
Giulio Maccauro (Rome, Italy)
Bruno Magnan (Verona, Italy)
Alessio Maiello (Turin, Italy)
Sergio Mapelli (Milan, Italy)
Ignazio Marcoccio (Brescia, Italy)
Marco Andrea Marino (Verona, Italy)
Marcos Almeida Matos (Salvador de Bahia, Brasil)
Cyril Mauffrey (Birmingham, UK)
Giovanni Merolla (Cattolica, Italy)
Jane Christiane Messina (Milan, Italy)
Maria Teresa Miscione (Bologna, Italy)
Giovanni Monteleone (Rome, Italy)
Thomasz Nizegorodcew (Rome, Italy)
Angela Notarnicola (Bari, Italy)
Matthew E. Oetgen (New Haven, CT, USA)
Alessia Pagnotta (Rome, Italy)
Paolo Paladini (Cattolica, Italy)
Vito Pavone (Catania, Italy)
Alessio Pedrazzini (Parma, Italy)
Giovanni Peretti (Milan, Italy)
Sandra Pfanner (Florence, Italy)
Franco Pleitavino (Genoa, Italy)
Enrico Pola (Rome, Italy)
Michele Rampoldi (Rome, Italy)
Bhagwat Singh Rao (Ahmedabad, India)
Enrico Rebuzzi (Oderzo, Italy)
Dario Regis (Verona, Italy)
Olav Reikerås (Oslo, Norway)
Stefano Rigotti (Negrar, Italy)
Giovanni Risitano (Messina, Italy)
E. Carlos Rodriguez-Merchan (Madrid, Spain)
Carlo L. Romanò (Milan, Italy)
Raffaele Russo (Naples, Italy)
Marco Saporito (Cattolica, Italy)
Alfredo Schiavone Panni (Campobasso, Italy)
Celeste Scotti (Milan, Italy)
Hamid Reza Shemshaki (Isfahan, Iran)
Nagraj Shetty (Raipur, India)
Saurabh Singh (Varanasi, India)
Giuseppe Solarino (Bari, Italy)
Toshiya Tachibana (Nishinomiya, Japan)
Gianpaolo Torasso (Orbassano, Italy)
Pierluigi Tos (Turin, Italy)
Federico Trentani (Bologna, Italy)
Cosimo Tudisco (Rome, Italy)
Luca Vaienti (san Donato Milanese, Italy)
Francesca Vannini (Bologna, Italy)
Manish Kumar Varshney (New Delhi, India)
Michele Arcangelo Verdano (Parma, Italy)
Anne Vochteloo (Leiden, The Netherlands)
Piero Volpi (Rozzano, Italy)
Stefano Zaffagnini (Bologna, Italy)
Giovanni Zatti (Monza, Italy)

Open Access

This article is distributed under the terms of the Creative Commons Attribution License which permits any use, distribution, and reproduction in any medium, provided the original author(s) and the source are credited.

